# Exosomal non-coding RNAs: mediators of crosstalk between cancer and cancer stem cells

**DOI:** 10.1038/s41420-025-02726-z

**Published:** 2025-10-06

**Authors:** Shuangmin Wang, Jiaojiao Shu, Nuoxin Wang, Zhixu He

**Affiliations:** 1 Department of Immunology, Zunyi Medical University, Zunyi, Guizhou Province China; 2 Key Laboratory of Cell Engineering of Guizhou Province, Affiliated Hospital of Zunyi Medical University, Zunyi, Guizhou Province China; 3 Collaborative Innovation Center of Chinese Ministry of Education, Zunyi Medical University, Zunyi, Guizhou Province China; 4 The Clinical Stem Cell Research Institute, Affiliated Hospital of Zunyi Medical University, Zunyi, Guizhou China; 5 Department of Pediatric Hematologic Oncology, Affiliated Hospital of Guizhou Medical University, Guiyang, Guizhou China

**Keywords:** Cancer, Cancer stem cells

## Abstract

Current advances in oncology have recognized two distinct cell subpopulations in tumors that include (1) a rare subpopulation, cancer stem cells (CSCs), which is considered to be the “seed” of the tumor, with therapy-resistant properties and as key drivers of tumor aggressiveness, and (2) the remaining bulk one, non-CSCs, all differentiated from the CSCs. Within the tumor microenvironment (TME), exosomes secreted by either CSCs or non-CSCs, containing multiple biomolecular cargos, mediate communication between both of the tumor cell subpopulations and play a vital role in promoting tumor progression. Specifically, a class of biomolecular cargo, non-coding RNAs (ncRNAs) that do not code for proteins during translation, has recently been highlighted to be a key participant in oncobiological processes. To comprehensively illuminate the mechanism of exosomal ncRNAs in mediating bidirectional communication between CSCs and differentiated tumor cells within the TME, we systematically analyzed the state-of-the-art literature from PubMed on this topic. It is revealed that: (1) Non-CSC exosomal ncRNAs enhance CSC stemness via upregulating stemness marker expression and activating stemness-reinforcing signaling pathways; (2) CSC-derived exosomal ncRNAs reciprocally mediate tumor progression by enhancing stemness, metastasis, angiogenesis, chemoresistance, and immune suppression of non-CSCs; (3) These tumor-derived exosomal ncRNAs possess the potentials as liquid biopsy biomarkers for early metastasis detection, and treatment targets or drug delivery systems for precision cancer therapy. It is therefore concluded that exosomal ncRNAs serve as critical communication bridges within TME, creating a self-reinforcing tumor-promoting loop, and therapeutically targeting exosomal ncRNAs could disrupt the crosstalk between CSCs and non-CSCs to delay the tumor progression. These findings provide a framework for developing combinatorial strategies against therapy-resistant malignancies.

## Facts

- The ncRNA within exosomes plays a pivotal role in maintaining the stemness of cancer stem cells (CSCs) and facilitating tumor progression.
- The deregulated expression of ncRNA in exosomes in tumors holds potential as a promising diagnostic and prognostic biomarker, as well as a therapeutic target, exhibiting tremendous potential in clinical applications.
- Strategies targeting tumor cells and CSCs, such as engineered specific exosomes and combined immunotherapy, are currently in the preclinical trial phase.

## Open questions

- What are the specific mechanisms that lead to the dysregulation of ncRNA in exosomes?
- What are the mechanisms by which exosomal ncRNA promotes the stemness of cancer stem cells (CSCs) and tumor progression?
- How can exosomes more efficiently load and deliver ncRNA to achieve clinical translation?

## Introduction

Cancer remains a leading global health threat, with over 20 million new cases and 9.7 million deaths reported worldwide in 2022. While therapeutic advances in surgery, radiotherapy, and targeted immunotherapy have significantly improved patient survival, critical challenges persist in clinical management due to high recurrence rate, metastatic propensity, and therapeutic resistance [1, 2]. Emerging evidence implicates cancer stem cells (CSCs) as key drivers of tumor aggressiveness [3]. CSCs, as therapy-resistant subpopulations in tumors, exhibit self-renewal capacity, multilineage differentiation potential, and remarkable adaptability to the tumor microenvironment (TME). Through cytokine/exosome secretion, CSCs mediate tumor heterogeneity and TME remodeling, ultimately fostering therapeutic evasion [4]. Exosomes have recently emerged as pivotal mediators of intercellular communication within the TME, with their non-coding RNAs (ncRNAs) cargo playing critical regulatory roles in tumor progression and stemness maintenance through gene silencing and epigenetic modifications [5]. For instance, circZFR may function as a molecular sponge for miR-3127-5p, sequestering it and thereby inhibiting its activity, which leads to the indirect upregulation of RTKN2 expression, ultimately activating downstream signaling pathways that promote the proliferation and migration of colorectal cancer cells [6], while lung CSC-derived exosomal long non-coding RNA (lncRNA) Mir100hg activates H3K14 lactylation to potentiate metastatic activity in non-CSCs [7]. Notably, current literature reviews predominantly focus on unilateral exosomal functions of either CSCs or non-CSCs, leaving bidirectional regulatory mechanisms mediated by exosomal ncRNA cross-talk between these populations not fully characterized [8]. Furthermore, the therapeutic potential of targeting specific exosomal ncRNAs - whether as molecular targets or drug delivery vehicles - remains underexplored [9]. This review systematically investigates the regulatory interplay between CSC-and non-CSC-derived exosomal ncRNAs within the TME, emphasizing their regulatory roles in tumor progression. We also critically evaluate the diagnostic and therapeutic potential of these molecules, proposing their utility as precision biomarkers for prognosis prediction, novel therapeutic targets for personalized interventions, and engineered delivery systems for targeted therapy.

## Cancer stem cell

CSCs are a subpopulation of tumor cells often referred to as the “seeds” of cancer. They possess the ability to self-renew, differentiate, and initiate tumor formation [10]. CSCs were first identified in 1997 by Bonnet and Dick in leukemia, where CD34^+^CD38^−^ populations were termed leukemia stem cells [11]. In recent years, CSCs have been identified and isolated in various solid tumors, including breast cancer [12], brain cancer [13], liver cancer [14], gastric cancer [15], colorectal cancer [16], ovarian cancer [17], prostate cancer [18], and so forth. Although CSCs account for only about 0.01%–2% of the tumor population, they play a crucial role in cancer initiation, progression, metastasis, and recurrence [19]. Additionally, CSCs play a key role in drug resistance due to their often dormant state and the presence of multiple drug-resistant molecules. For instance, Mare et al. [20] discovered that pre-treatment of MCF-7 breast cancer cells with paclitaxel enhanced their ability to form mammospheres, indicating that tumor stem cells can resist drug treatment. Furthermore, increasing evidences suggest that, in addition to their strong self-renewal capacity, CSCs exhibit “plasticity.” This means that under the influence of both intrinsic and extrinsic factors, they can differentiate into various cancer cell subtypes that adapt to environmental changes, significantly promoting tumor growth, metastasis, and resistance to therapy. This plasticity is also considered one of the main contributors to tumor heterogeneity [21]. The origin of CSCs remains unclear, and several hypotheses have been proposed: (1) The fusion of normal stem cells with cancer cells [22]; (2) The transformation of normal stem cells, in which genetic mutations, environmental factors, or other genetic alterations induce oncogenic mutations and excessive self-renewal capacity [23]; (3) Genetic instability, which may arise from chromosomal changes (including gains and losses of chromosomes) or molecular alterations, to affect progenitor cells and even differentiated cells [24]; (4) The influence of the cellular microenvironment, where specific changes and stimuli trigger the clonal expansion of stem cells and differentiated cells. This process leads to the production of inflammatory cytokines/chemokines, causing differentiated cells to de-differentiate into CSCs [25]. Currently, in vitro tumor sphere formation and in vivo limiting dilution assays are considered the gold standards for identifying CSCs. However, numerous studies have shown that the expression of CSC surface markers and the regulation of related pathways controlling self-renewal and survival can also serve as indicators of CSC specificity [26]. Common markers of CSCs include CD44, CD133, OCT4, EpCAM, and aldehyde dehydrogenase (ALDH), among others. However, the surface markers may vary across different types of cancers. For example, breast cancer stem cells express CD49f, CD61, and epithelial-specific antigen (ESA) [27], while pancreatic CSCs exhibit markers such as CD44/CD24 and ESA [28]. Additionally, CSCs can activate multiple signaling pathways, including Wnt/β-catenin, Notch, and PI3K/AKT/mTOR. These pathways, involving both genetic and epigenetic modifications, play a crucial role in maintaining CSC stemness and regulating key processes, such as cell survival, growth, self-renewal, and differentiation [29].

## Functions and characteristics of exosomes and exosomal ncRNAs

### Introduction to exosomes

Exosomes are extracellular vesicles with a diameter of 30–150 nm, secreted by various cell types and widely present in biological fluids. They play a key role in mediating intercellular communication and are involved in multiple stages of cancer, including tumor initiation, progression, metastasis, drug resistance, and treatment response. Additionally, exosomes contribute to immune responses and tissue repair, playing significant roles in various diseases [30]. The functional capabilities of exosomes mainly result from their rich content of bioactive molecules, including proteins, lipids, and nucleic acids (DNA, mRNA, and ncRNAs) [31]. Proteins such as CD9, CD63, and CD81 enhance exosome recognition and targeting abilities, playing vital roles in signal transmission and cargo transport. Nucleic acids facilitate intercellular communication and gene regulation. The lipid bilayer membrane, which includes cholesterol, sphingolipids, ceramides, and phosphatidylcholine, provides exosomes with stability and protection, while also determining their interactions with other cells [32]. The composition of these substances may vary depending on the source cell and isolation method, and their specific assembly and packaging endow exosomes with distinct functions [33].

**Fig. 1:**
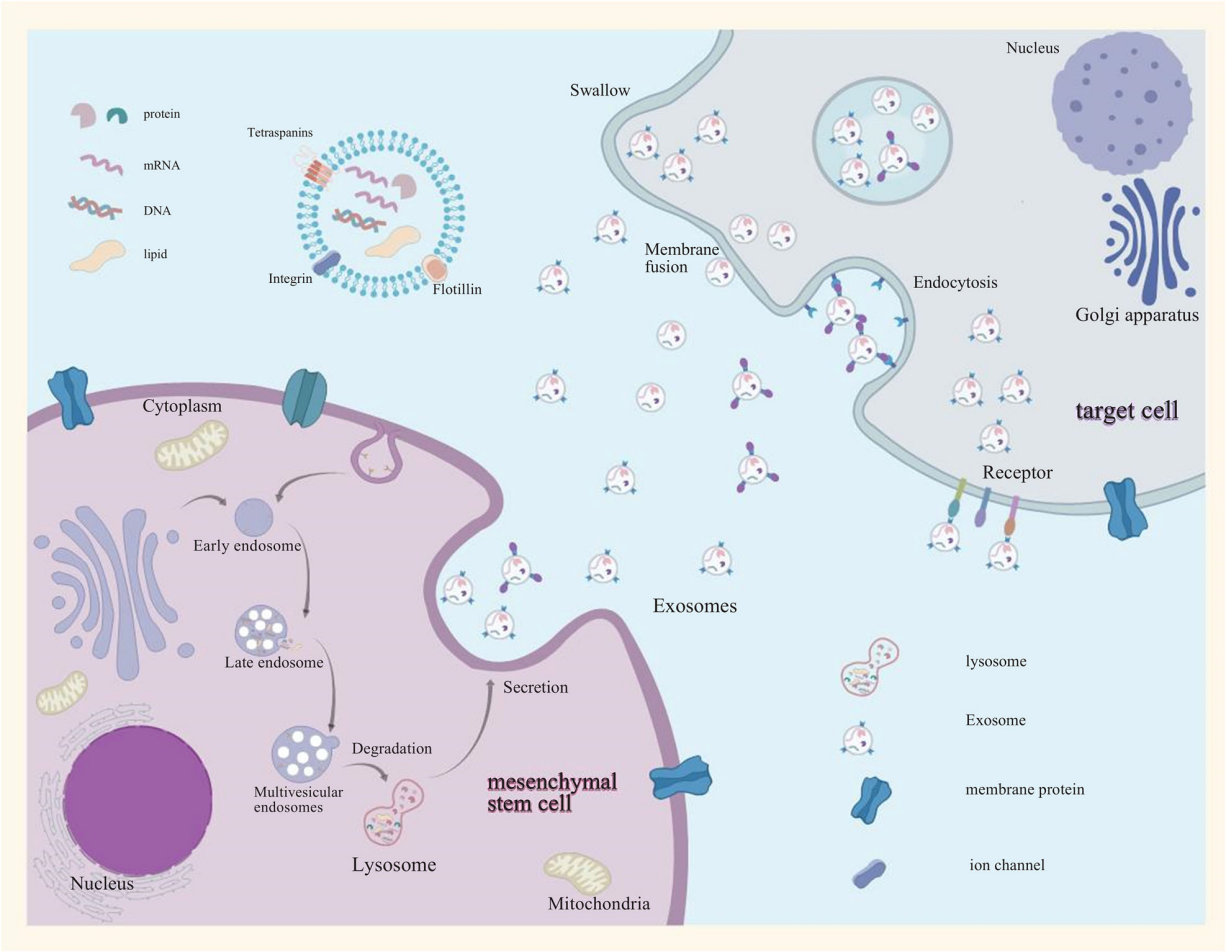
The formation process of exosomes, as well as their release and uptake mechanisms. Exosomes are initially formed through the invagination of the plasma membrane, leading to the creation of early endosomal vesicles. These early endosomal vesicles undergo further invagination to form late endosomes, which accumulate mRNA, proteins, lipids, and other materials through various complex mechanisms, eventually forming multivesicular bodies (MVBs). MVBs can either fuse with lysosomes for degradation or release exosomes through fusion with the plasma membrane. Exosomes can be taken up by target cells through various mechanisms, including direct membrane fusion, internalization via endosome encapsulation followed by membrane fusion, membrane protrusion and phagocytosis, or receptor-ligand interactions.

Exosome formation begins with the inward budding of the plasma membrane, leading to the creation of early endosomes [34]. Through a variety of complex mechanisms, including both Endosomal Sorting Complex Required for Transport (ESCRT)-dependent and ESCRT-independent pathways, proteins, lipids, and nucleic acids accumulate in the endosome to form intraluminal vesicles (ILVs), which further mature into multivesicular bodies (MVBs) [35]. MVBs can either fuse with lysosomes for degradation or with the plasma membrane to release exosomes [36]. The transport and fusion of MVBs with the plasma membrane are regulated by the Rab GTPase family (such as Rab27a/b) and Rab-interacting lysosomal proteins [37]. When interacting with target cells, exosomes can be internalized via several mechanisms, including direct membrane fusion, membrane invagination followed by endocytic uptake, membrane protrusion and engulfment, or receptor-ligand binding [38]. The formation process of exosomes, as well as their release and uptake mechanisms, is illustrated in Fig. 1.

Current methodologies for exosome isolation leverage diverse principles, including size exclusion, charge difference, and affinity-based interactions. Established techniques encompass the gold-standard ultracentrifugation protocol, scalable ultrafiltration approaches utilizing molecular weight cutoffs, size-exclusion chromatography, polymer-induced precipitation, high-purity immunoaffinity capture, microfluidics-enabled platforms, and membrane-based affinity systems [39]. However, these conventional methods often involve complex workflows, lengthy processing times, and yield suboptimal purity levels, limiting their scalability for industrial-scale production. Recent advances advocate for systematic integration of isolation and characterization technologies to achieve synergistic efficiency gains. A novel “isolation-characterization” hybrid platform enables rapid optimization of process parameters through iterative feedback loops, facilitating simultaneous enhancement of exosome yield and purity [40]. Specifically, multistage centrifugation protocols incorporating gradient speed/time profiles can be implemented in ultracentrifugation workflows, while immunomagnetic beads functionalized with target-specific antibodies allow selective exosome enrichment. Subsequent characterization via transmission electron microscopy (TEM) utilizes focused electron beams to generate high-resolution images of exosome ultrastructure post-electromagnetic lens focusing. Complementary cryo-scanning electron microscopy (Cryo-SEM) permits real-time morphological analysis and particle size distribution measurements under near-physiological conditions, providing critical process optimization metrics. This integrated technological framework not only streamlines isolation efficiency but also establishes closed-loop quality control throughout the manufacturing continuum [41].

### Classification and functions of non-coding RNAs

Non-coding RNAs (ncRNAs), a class of RNA molecules that do not directly encode proteins, have gained increasing attention in recent years. ncRNAs regulate gene expression at the transcriptional, post-transcriptional, and translational levels, and play crucial roles in processes such as the cell cycle and cell differentiation [42]. Currently, the most studied ncRNAs are classified based on their size into microRNAs (miRNAs), long non-coding RNAs (lncRNAs), and circular RNAs (circRNAs). MiRNAs are approximately 20–24 nucleotides long and primarily regulate gene expression at the post-transcriptional level by guiding the RNA-induced silencing complex (RISC) to degrade target mRNAs or inhibit their translation [43]. Studies have shown that exosome-derived miRNAs act as paracrine agonists of Toll-like receptors (TLRs) and are key regulators of tumor initiation and cancer progression [44]. Additionally, miRNAs can modulate the tumor microenvironment, influencing the extracellular matrix (ECM) and the activation and recruitment of the immune system [45]. LncRNAs are typically longer than 200 nucleotides and have little to no protein-coding capacity. They can participate in gene expression regulation at the epigenetic, transcriptional, and post-transcriptional levels [46]. For instance, lncRNAs can function as “molecular sponges,” reducing the inhibitory effects of miRNAs on target genes [47], bind to transcription factors to regulate downstream gene expression, and participate in chromatin remodeling to alter its structure [48, 49]. CircRNAs, typically ranging in length from 200 to 1200 nucleotides, are circular molecules formed through back-splicing of pre-mRNA, which is synthesized by RNA polymerase II along the DNA template strand. The unique structure of circRNAs, lacking a 5′ cap and 3′ polyadenine tail, makes them resistant to degradation by nucleases, resulting in greater stability compared to linear RNAs [50]. CircRNAs can serve as important regulatory factors in gene expression through various mechanisms, including acting as sponges for miRNAs, modulating gene transcription and protein translation, and interacting with RNA-binding proteins [51–53].

### Sorting mechanisms of non-coding RNAs in exosomes

It is widely accepted that the process of sorting specific ncRNAs into exosomes is primarily influenced by lipids and proteins. In a study by Kosaka et al., neutral sphingomyelinase 2 (nSMase2) was identified as the first molecule responsible for sorting miRNAs into exosomes, with its expression level positively correlating with the amount of miRNA present in exosomes [54]. Certain proteins can recognize and bind to specific ncRNA sequences, thereby regulating the selective incorporation of these ncRNAs into exosomes. For instance, miRNAs contain a conserved sequence called the EXO motif, and hnRNPA2B1 can bind to this sequence through its EXO motif, controlling the loading of specific subsets of miRNAs into exosomes. Notably, hnRNPA2B1 in exosomes can be sumoylated, a post-translational modification that further regulates the binding of hnRNPA2B1 to miRNAs, thus affecting their sorting [55]. Argonaute 2(AGO2), a key component of the miRNA-induced silencing complex (miRISC) and a carrier for miRNAs, also plays a critical role in the packaging and transfer of miRNA into exosomes [56]. In addition to miRNAs, the sorting of circRNAs in exosomes is also regulated by a range of RNA-binding proteins, such as ribosomal proteins and elongation factors, which can influence circRNA sorting either directly or indirectly [57]. However, the underlying mechanisms of ncRNA sorting are not yet fully understood and require further investigation.

## Role of non-CSC-derived exosomal ncRNAs in CSC stemness

The TME is a complex, homeostatic milieu composed of CSCs, non-CSC tumor cells, immune cells, cancer-associated fibroblasts (CAFs), endothelial cells (ECs), the ECM, and various signaling molecules that regulate tumor progression [58]. Similar to normal stem cells, CSCs possess the ability to self-renew, replicate indefinitely, and differentiate into multiple cell types. Exosomal ncRNAs derived from non-CSC cells within the TME can promote the self-renewal capacity of CSCs and maintain their stemness by regulating the expression of genes or signaling pathways associated with stemness maintenance. Table 1 summarizes the mechanism of exosomal ncRNAs from non-CSC sources to support the maintenance of CSC stemness by modulating stemness-related genes and pathways.

**Table 1:** Exosomal ncRNAs derived from non-CSCs promote CSC stemness.

Exosomal ncRNA	Source	Function	Mechanism	References
LncRNA-ROR	Pancreatic cancer cells	Promotes CSC stemness and proliferation	Acts as a ceRNA to regulate microRNA function, upregulates the expression of stemness markers such as OCT4, Sox2, and Nanog.	[53]
miR-138-5p	Oral squamous carcinoma cells	Promotes CSC stemness, growth, and metastasis	Interacts with Np63, upregulates stemness markers such as Sox2, KLF4, NOTCH1, and CD44.	[54]
lncRNA MALAT1	Colorectal cancer cells	Promotes CSC stemness, increases CSC population	Regulates the miR-20b-5p/OCT4 axis, upregulates stemness markers such as OCT4 and Nanog	[55]
lncRNA MACC1-AS1	Nasopharyngeal carcinoma cells	Promotes CSC stemness and enhances tumorigenic potential	Antagonizes miR-145 activity, targeting Smad2, and upregulates the expression of Sox9 and OCT4	[57]
miR-328-3p	Ovarian cancer cells	Maintains and enhances CSC stemness characteristics and activity	Directly targets and suppresses the expression of DDB2	[58]
miR-155	MSC	Enhances the stemness and drug resistance of multiple myeloma CSC	Upregulates the expression of OCT4, Nanog, and drug resistance-associated proteins	[60]
miR-142-3p	MSC	Promotes CSC stemness phenotype and tumor growth |	Upregulates CD133 and Lgr5, while inhibiting Numb expression	[61]
circHIF1A	Fibroblast-associated cells	Promotes CSC stemness	Regulates CD44 expression and acts as a sponge for miR-580-5p	[63]
LncRNA PKMYT1AR	Non-small cell lung cancer	Promotes tumorigenesis and maintains CSC stemness	Activates the Wnt/β-catenin signaling pathway	[66]
miR-1275	Lung adenocarcinoma cells	Maintains CSC phenotype and promotes tumor progression	Activates the Wnt/β-catenin and Notch pathways	[67]
miR-454	Breast cancer cells	Maintains CSC phenotype and promotes tumor progression	Activates the PRRT2/Wnt pathway	[68]
miR-92a-3p	Fibroblast-associated cells	Promotes tumor growth and stemness in liver cancer	Inhibits AXIN1 expression and activates the Wnt/β-catenin signaling pathway	[69]
miR-21, miR-27b, miR-329	Oral squamous cell carcinoma	Alter stemness-related characteristics such as EMT or invasiveness	Regulate the Wnt signaling pathway	[70]
miR-600	Ovarian cancer cells	Promotes tumor stemness and metastasis	Increases the expression level of NOTCH1 and enhances Notch pathway activity	[72]
miR-200c	Ovarian cancer cells	Promotes the development of CSC stemness and drug resistance	Inhibits PTEN expression and activates the PI3K/AKT pathway	[73]
miR-146a-5p	Fibroblast-associated cells	Enhances CSC characteristics and drug resistance	Activates the STAT3 and mTOR signaling pathways	[75]
miR-378a-3p miR-378d	Breast cancer cells	Promote CSC stemness and drug resistance	Activate the EZH2/STAT3 pathway	[76]

### Exosomal ncRNAs from non-CSCs upregulate stemness markers in CSCs

Exosomal ncRNAs play a critical role in maintaining the stemness characteristics of CSCs by regulating the expression of key stemness-related genes, thereby promoting their self-renewal and proliferation. In recent years, numerous studies have shown that exosomes from tumors, such as pancreatic cancer [59], oral squamous cell carcinoma [60], colorectal cancer [61], osteosarcoma [62], and nasopharyngeal carcinoma [63], can upregulate stemness markers such as CD44, CD133, OCT4, Sox2, and Nanog through ncRNAs. This upregulation helps sustain the stemness of CSCs while also enhancing their invasive, migratory abilities and chemotherapy resistance. Additionally, ncRNAs can promote CSC stemness by downregulating proteins that suppress CSC activity. For example, Srivastava et al. found that in ovarian cancer, miR-328-3p directly targets and suppresses the expression of DDB2, a protein that limits the CSC population and inhibits its activity, thereby maintaining and enhancing the stemness and activity of CSCs [64]. In addition to tumor cells, mesenchymal stem cells (MSCs) within the TME also contribute to the maintenance of CSC stemness through exosomal ncRNAs. MSCs, with their high self-renewal capacity and multidirectional differentiation potential, release exosomes with high plasticity and adaptability based on the surrounding environment. Studies have shown that bone marrow-derived mesenchymal stem cells (BM-MSCs) can regulate stemness characteristics of various types of CSCs via exosomal ncRNAs [65]. MiR-155 within exosomes derived from BM-MSCs can significantly inhibit cell apoptosis, promote cell proliferation, and upregulate the expression of stemness-related markers (such as OCT4 and Nanog) as well as drug-resistance-associated proteins. Consequently, it enhances the stemness and chemotherapy resistance of multiple myeloma (MM) cells [66]. Li et al. found that BM-MSC-derived exosomes contain miR-142-3p, which upregulates the expression of CD133 and Lgr5 while inhibiting the expression of Numb. This activates the Notch signaling pathway and promotes the expression of downstream target genes, increasing the CSC population and enhancing their stem cell-like characteristics [67]. As an important component of the TME, CAFs also regulate CSC stemness phenotypes by secreting exosomes containing ncRNAs. Deng et al. found that co-culturing CAFs with pancreatic cancer cells under hypoxic conditions increased the protein expression of CD44, CD133, OCT4, and Sox2, leading to an increase in CSC numbers and significantly enhancing tumor resistance [68]. Zhan et al. demonstrated that exosomes from CAFs transfer circHIF1A to breast cancer cells, where it functions as a sponge for miR-580-5p, thereby regulating CD44 expression and promoting the development of tumor stemness [69]. Moreover, exosomes from immune cells in TME can also contribute to CSC stemness by transferring ncRNAs that modulate immune signaling and immune responses. One study found that exosomes secreted by M2 macrophages are rich in miR-27a-3p, which significantly reduces the expression of thioredoxin-interacting protein (TXNIP) in liver cancer, thereby supporting the maintenance and activation of liver CSCs [70].

### Exosomal ncRNAs from non-CSCs activate stemness-related signaling pathways in CSCs

Exosomes participate in regulating the stemness and tumorigenicity of CSCs by modulating various stemness-related signaling pathways. Among these, the Wnt signaling pathway plays a central role in maintaining stem cell properties. Aberrant activation of this pathway can significantly promote tumorigenesis and enhance CSCs’ self-renewal and differentiation capabilities [71]. A study by He et al. demonstrated that the lncRNA PKMYT1AR interacts with miR-485-5p to activate the Wnt/β-catenin pathway, thereby promoting the maintenance of stemness in CSCs in non-small cell lung cancer (NSCLC) and contributing to tumorigenesis [72]. In lung adenocarcinoma (LUAD), upregulated miR-1275 simultaneously activates both the Wnt/β-catenin and Notch signaling pathways, maintaining a stem cell-like phenotype and significantly advancing LUAD progression [73]. Furthermore, exosomal microRNA-454 released by breast cancer cells maintains the biological characteristics of CSCs in ovarian cancer by activating the PRRT2/Wnt axis [74]. In oral squamous cell carcinoma, miRNAs such as miR-21, miR-27b, and miR-329 can regulate the Wnt signaling pathway at different levels, thereby altering stemness-related characteristics such as epithelial-mesenchymal transition (EMT) or invasiveness [75, 76]. Aberrant activation of the Notch signaling pathway is also closely associated with CSC proliferation and drug resistance. Research conducted by Sun et al. has revealed that the Notch signaling pathway plays a crucial regulatory role in the proliferation, stemness maintenance, and invasion processes of NSCLC cells. Its aberrant activation can lead to the development of chemotherapy resistance in NSCLC cells through various mechanisms, such as sustaining the stem cell-like properties of cancer cells or modulating the tumor microenvironment [77]. Studies have shown that miR-600, by inhibiting the expression of KLF6, promotes the binding of transcriptional activators to the promoter region, thus upregulating the expression of Notch1, enhancing Notch pathway activity, and ultimately promoting stemness maintenance and metastatic potential in ovarian cancer cells [78]. Furthermore, exosomal ncRNAs can also influence CSC stemness by regulating other signaling pathways. For example, miRNAs can facilitate the activation of the PI3K/Akt signaling pathway. By modulating the activity of downstream key transcription factors, they enhance the self-renewal capacity of CSCs and sustain their differentiation potential. Consequently, this process drives the maintenance of stemness in ovarian cancer [79] and endometrial cancer [80]. Zhuang et al. experimentally found that exosomes containing miR-146a-5p secreted by CAFs in urothelial bladder cancer can enhance CSC stemness and drug resistance by activating the STAT3 and mTOR signaling pathways [81]. In breast cancer, exosomes secreted by the tumor contain miR-378a-3p and miR-378d, which target Numb and DKK3 to activate the EZH2/STAT3 pathway, thereby promoting CSC stemness and chemoresistance [82].

## Role of CSC-derived exosomal ncRNAs in tumor progression

**Fig. 2:**
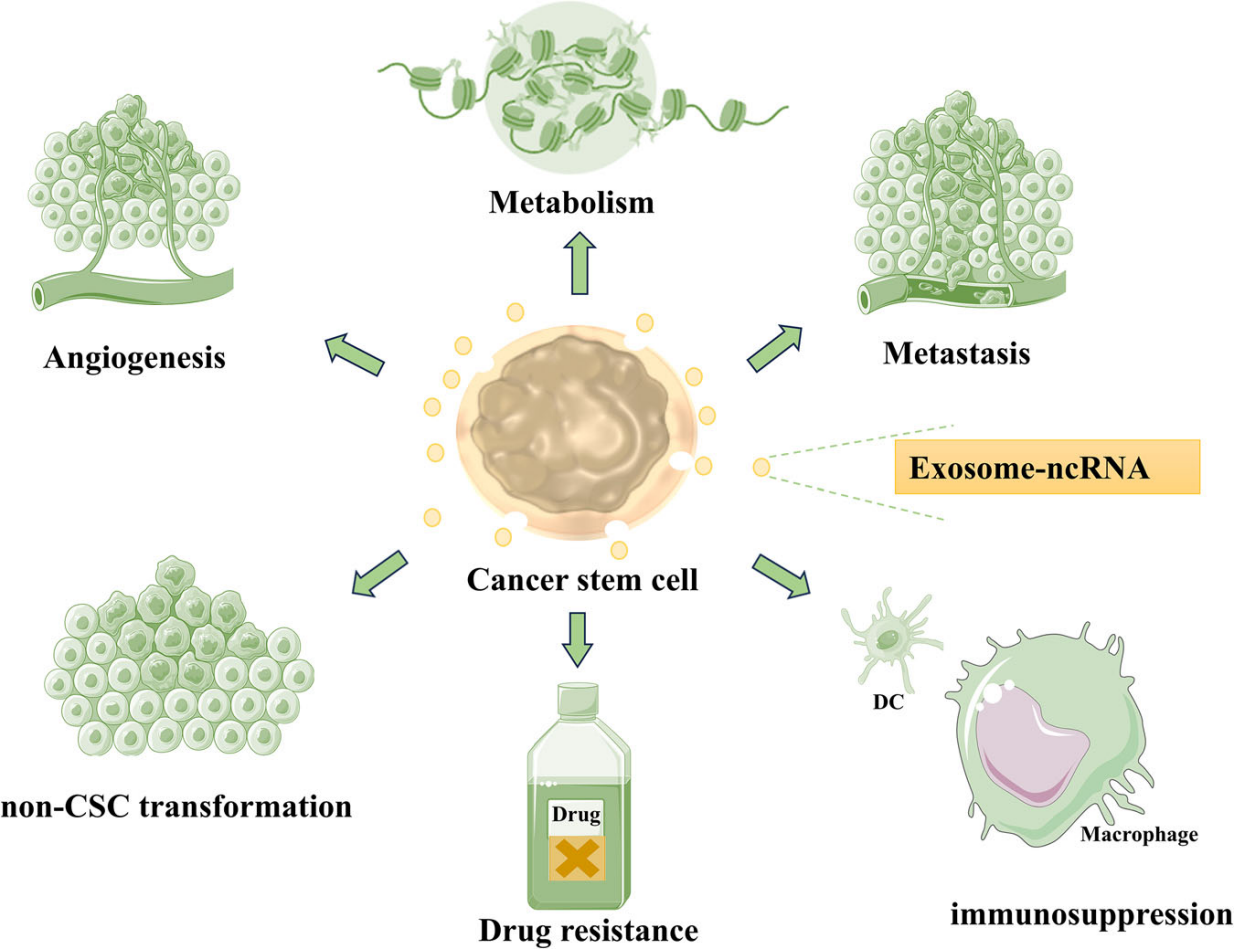
Role of CSC-derived exosomal ncRNAs in tumor progression. Exosomes derived from CSCs promote the stemness conversion of non-CSCs, accelerate tumor metastasis, induce angiogenesis, enhance drug resistance, and tumor metabolic reprogramming, while also contributing to the establishment of a tumor-supportive and immunosuppressive microenvironment.

Besides, exosomal ncRNAs derived from non-CSCs play a crucial role in CSCs; exosomal ncRNAs secreted by CSCs can inversely promote the stemness conversion of non-CSCs, accelerate tumor metastasis, induce angiogenesis, enhance drug resistance, and reprogram tumor metabolism as well. These exosomal ncRNAs also contribute to the creation of a tumor-favorable and immune-suppressive microenvironment, as shown in Fig. 2. Table 2 summarizes the promoting effects and the specific mechanisms of exosomal ncRNAs secreted by CSCs in tumors.

**Table 2:** Exosomal ncRNAs derived from CSCs regulate tumor progression.

Exosomal ncRNA	Source	Function	Mechanism	Reference
miR-146a-5p	Colorectal CSCs	Promotes asymmetric division and maintains the stemness of CSCs	Inhibits the expression of Numb and activates the Wnt/β-catenin signaling pathway	[78]
circRNA ABCC1	Colorectal CSCs	Promotes tumor stemness and metastasis	Activates the Wnt/β-catenin signaling pathway	[79]
lncRNA FMR1-AS1	Esophageal CSCs	Maintains CSC homeostasis and promotes tumor stemness	Activates the TLR7-NFκB pathway and upregulates the expression of c-Myc in recipient cells	[80]
lncRNA DOCK9-AS2	Thyroid CSCs	Promotes tumor stemness	Acts as a sponge for miR-197 and activates the Wnt/β-catenin signaling pathway	[81]
circ-ZEB1, circ-AFAP1	Liver CSCs	Promotes tumor stemness and metastasis	Increases the expression of stemness marker CD133 and downregulates the expression of E-cadherin and EpCAM	[83]
miR-210-3p	Lung CSCs	Promotes EMT and metastasis	Upregulates the expression of EMT-related markers in lung cancer cells and downregulates the expression of E-cadherin	[88]
lncRNA MALAT1, linc-ROR	Thyroid CSCs	Promotes EMT, invasion, and metastasis	Upregulates the expression of EMT-related markers	[89]
miR-19b-3p	Renal CSCs	Promotes EMT and lung metastasis	Downregulates PTEN expression and induces EMT	[90]
miR-21,lncTuc339,lncHEIH P13K	Liver CSCs	Induces EMT and enhances tumor invasion and metastasis	Affects the mRNA levels of Bcl2, TGFβ1, NFκB, VEGF, and MMP9, and activates corresponding signaling pathways	[91]
miR-4535	Melanoma CSCs	Enhances tumor metastasis	Inhibits the MPC autophagy pathway	[94]
miR-26a	Glioblastoma CSCs	Promotes angiogenesis	Targets and inhibits PTEN expression, activating the PTEN/PI3K/AKT signaling pathway	[97]
miR-21	Glioblastoma CSCs	Promotes angiogenesis	Upregulates the expression of VEGF	[98]
lncRNA H19	Liver CSCs	Promotes angiogenesis	Regulates ECs, promoting an angiogenic phenotype and intercellular adhesion	[99]
miR-21-5p	Oral Squamous CSCs	Promotes angiogenesis, tumor stemness, and drug resistance	Activates the β-catenin/mTOR/STAT3 signaling pathways and promotes the transformation of cancer-associated fibroblasts (CAFs)	[101]
miR-210	Pancreatic CSCs	Promotes gemcitabine resistance in tumors	Facilitates drug efflux and activates the mTOR signaling pathway	[108]
miR-30b-3p	Glioma CSCs	Promotes tumor resistance to temozolomide	Targets RHOB, leading to decreased apoptotic capability	[107]
miR-155	Breast CSCs	Promotes resistance to doxorubicin and paclitaxel	Inhibits the expression of TGF-β, C/EBP-β, and FOXO3a	[110]
miR-17-5p	Colorectal CSCs	Promotes immune evasion	Targets SPOP and overexpresses PD-L1, inhibiting antitumor immunity in colorectal cancer	[114]
miR-20a	Breast CSCs	Promotes immune evasion	Downregulates the expression of NKG2D ligands, allowing escape from NK cell-mediated killing	[119]
lncRNA UCA1	Oral Squamous CSCs	Promotes immune suppression	Binds with miR-134 to target LAMC2 and regulates the PI3K/AKT pathway in macrophages	[121]
miR-146a	Colorectal CSCs	Promotes immune suppression	Reduces the number of tumor-infiltrating CD8+ T cells	[122]
lncRNA MALAT1	Glioma CSCs	Promotes immune suppression	Induces microglial cells stimulated by LPS to secrete IL-6 and TNF-α	[123]
circCARM1	Breast CSCs	Promotes tumor metabolic reprogramming	Acts as a sponge for miR-1252-5p, thereby regulating the expression of the PFKFB2 gene and promoting glycolysis	[127]
lncRNA Mir100hg	Lung CSCs	Promotes tumor metabolic reprogramming	Targets miR-15a-5p and miR-31-5p in tumor cells, enhancing glycolytic activity	[128]
lncRNA Mir100hg	Melanoma CSCs	Promotes tumor metabolic reprogramming	Targets the expression of miR-16-5p and miR-23a-3p, enhancing the glycolytic process	[129]
lncRNA ROLLCSC	Lung CSCs	Promotes tumor metabolic reprogramming	Acts as a competitive endogenous RNA (ceRNA) for miR-5623-3p and miR-217-5p, targeting lipid metabolism pathways	[130]
lncRNA H-GSC	Glioma CSCs	Promotes tumor metabolic reprogramming	Regulates the MZF1/c-Myc/HIF-1α axis, significantly enhancing tumor cell proliferation and glycolytic metabolism	[131]

### Exosomal ncRNAs from CSCs promote the transformation of non-CSCs

The primary function of CSCs is to utilize paracrine factors to promote tumorigenesis and enhance the stemness characteristics of non-CSCs. A growing body of evidence suggests that exosomes derived from CSCs contain higher levels of stemness markers and proteins, which can be transferred to non-CSCs to enhance their stemness [83]. Moreover, the ncRNAs within exosomes play an essential role in regulating CSC differentiation and the dedifferentiation of tumor cells. These ncRNAs guide cells toward diverse tumor cell types, thereby increasing tumor heterogeneity and maintaining CSC homeostasis. One study found that miR-146a-5p, by inhibiting the expression of Numb, affects the distribution of PKH26-labeled vesicles in colorectal CSCs, suggesting that this regulation may be one of the mechanisms driving asymmetric division in CSCs [84]. A further finding from Zhao et al.‘s [85] study reveals that exosomes secreted by CD133^+^ colorectal CSCs carry circRNA ABCC1. This circRNA activates the Wnt/β-catenin pathway, enhancing the stemness phenotype and spheroid formation ability of non-colorectal CSCs, thereby promoting colorectal cancer progression. Exosomes derived from esophageal CSCs contain the lncRNA FMR1-AS1, which binds to endosomal Toll-like receptor 7 (TLR7) and activates the NFκB signaling pathway. This activation promotes the expression of the stem cell-related gene c-Myc in non-CSCs, enhancing their stemness and contributing to tumor progression [86]. Additionally, exosomes derived from thyroid CSCs contain the lncRNA DOCK9-AS2, which activates the Wnt/β-catenin pathway, promoting stemness maintenance and malignant progression of the tumor [87]. At the same time, ncRNAs can promote the differentiation and metastasis of CSCs by regulating processes such as EMT. Exosomes released by pancreatic ductal adenocarcinoma (PDAC) carry the lncRNA Sox2ot, which binds to miR-200c and regulates the expression of Sox2, thereby enhancing the EMT process and stemness characteristics, and increasing the tumor’s metastatic and invasive capabilities [88]. Han et al. found that exosomes derived from liver CSCs contain circ-ZEB1 and circ-AFAP1, which significantly increase the expression of the stemness marker CD133. At the same time, these exosomal circRNAs decrease the expression levels of E-cadherin and epithelial cell adhesion molecule (EpCAM), which are associated with the EMT process. This suggests that the ncRNAs in exosomes not only promote cancer cell stemness and the EMT process but also facilitate the progression of hepatocellular carcinoma (HCC) [89].

### Exosomal ncRNAs from CSCs promote tumor metastasis

Tumor metastasis, as a key hallmark of malignant tumor progression, plays a crucial role in assessing patient prognosis. CSCs, with their ability for self-renewal and unlimited proliferation, serve as the foundation for tumor growth and metastasis. Furthermore, CSCs enhance tumor invasiveness and metastatic potential by differentiating into various types of tumor cells to adapt to the ever-changing microenvironment [90]. More importantly, ncRNAs in exosomes secreted by CSCs can significantly promote tumor metastasis and growth by inducing EMT and establishing pre-metastatic niches. These ncRNAs also regulate various components of the tumor microenvironment, creating a microenvironment that favors tumor metastasis [91]. EMT enables tumor cells to acquire mesenchymal characteristics, allowing them to more easily cross the basement membrane and blood vessel walls, enter the bloodstream or lymphatic system, and thereby increase their migratory and invasive capabilities [92]. Exosomal ncRNAs derived from CSCs can directly regulate EMT-related genes, triggering and accelerating the EMT process. For instance, exosomes from lung cancer CSCs, through miR-210-3p, upregulate the expression of EMT-related markers such as N-cadherin, vimentin, matrix metalloproteinase 9 (MMP9), and MMP-1, while downregulating the expression of E-cadherin, significantly promoting lung cancer metastasis [93]. Hardin et al. found that exosomes from thyroid cancer CSCs, which highly express lncRNA MALAT1, linc-ROR, and EMT markers, can induce an EMT phenotype when co-cultured with normal thyroid cells. These exosomes also facilitate the establishment of a niche in the TME and distant metastatic sites [94]. Wang et al. demonstrated that exosomes secreted by clear cell renal cell carcinoma (CCRCC) stem cells deliver miR-19b-3p to tumor cells, which reduces the expression of the tumor suppressor gene PTEN, induces EMT in tumor cells, and promotes metastasis [95]. Additionally, exosomes derived from liver cancer CSCs can upregulate the expression of ncRNAs such as miR-21, lncTuc339, lncHEIH, and P13K, influencing the mRNA levels of Bcl2, TGFβ1, NFκB, and MMP9. This activation of corresponding signaling pathways induces EMT and enhances tumor invasiveness and metastasis. However, exosomes derived from BM-MSCs can reverse the expression of the aforementioned molecules, suppress EMT phenotypic changes, and, to a certain extent, inhibit tumor metastasis [96]. Exosomal ncRNAs from CSCs also promote tumor metastasis through alternative pathways, such as regulating the tumor microenvironment, promoting angiogenesis, and modulating drug resistance. Studies have shown that exosomes from colorectal CSCs enhance the activity of neutrophils in the bone marrow, promoting tumor characteristics. The presence of active CSC signaling and an increase in tumor-infiltrating neutrophils suggest that neutrophils may participate in mediating EMT and maintaining the CSC niche [97]. He et al. demonstrated that exosomal miR-205 derived from ovarian cancer can promote metastasis by inducing angiogenesis through the PTEN-AKT pathway [98]. Recently, it has also been reported that exosomes from CSCs can promote tumor metastasis and colonization by inhibiting autophagy pathways. Liu et al. found that exosomes from melanoma CSCs deliver miR-4535 to low-metastatic melanoma cells, significantly enhancing the tumor’s metastatic ability by inhibiting their autophagy pathways [99].

### Exosomal ncRNAs from CSCs promote tumor angiogenesis

Angiogenesis, which provides tumors with nutrients and oxygen, is considered a critical factor in cancer progression [100]. Studies have shown that exosomes derived from CSCs can regulate the expression of angiogenic factors by interacting with ECs and stromal cells in the TME, thereby activating related signaling pathways to promote tumor vasculature formation [101]. Exosomes secreted by glioblastoma CSCs contain miRNAs that promote angiogenesis through different mechanisms. For instance, miR-26a can target and suppress the expression of PTEN, activating the PI3K/Akt signaling pathway, which enhances tumor growth and metastasis [102]. Additionally, miR-21 can upregulate the expression of vascular endothelial growth factor (VEGF), thereby promoting angiogenesis in ECs [103]. Exosomes from liver CSCs, which contain CD90^+^ cells, can also regulate ECs by delivering lncRNA H19, promoting an angiogenic phenotype, cell-cell adhesion, and influencing the tumor microenvironment [104]. Additionally, exosomal ncRNAs derived from CSCs can further promote angiogenesis by regulating the secretion of pro-angiogenic factors and inflammatory mediators within the TME. Under hypoxic conditions, exosomes derived from lung cancer cells carry miR-23a, which targets prolyl hydroxylase and tight junction protein ZO-1, promoting angiogenesis and modulating cancer progression [105]. Exosomes from oral cancer CSCs can enhance tumor angiogenesis and metastasis by upregulating the expression levels of miR-21-5p and transforming growth factor TGF-β1. This activates the β-catenin/mTOR/STAT3 pathway, converting normal fibroblasts into CAFs with enhanced carcinogenic potential [106]. Vera et al. observed that when exosomes secreted by cisplatin-treated ovarian CSCs were co-cultured with MSCs, the expression of interleukin-6 (IL-6), interleukin-8 (IL-8), and vascular endothelial growth factor A (VEGFA) increased, along with enhanced pro-tumor activity in MSCs. This finding suggests that intercellular interactions within the tumor microenvironment promote angiogenesis and matrix remodeling, thereby regulating tumor progression [107].

### Exosomal ncRNAs from CSCs promote tumor drug resistance

Tumor drug resistance is a major cause of malignant cancer progression and poor prognosis, with CSCs being considered a key factor in driving resistance. Increasing evidence suggests that exosomes derived from CSCs can deliver ncRNAs to non-CSC regions, thereby contributing to the development of chemotherapy resistance through various mechanisms, such as altering apoptotic pathways, enhancing drug efflux, and inducing drug resistance in chemotherapy-sensitive cells [108–110]. Exosomes from CSCs promote tumor drug resistance by reprogramming the cell cycle and apoptosis genes in recipient cells. Fornari et al. [111] experimentally demonstrated that exosomes derived from HCC cells carry miR-221, which directly targets caspase-3, inhibiting tumor cell apoptosis and increasing resistance to sorafenib in HCC cells. Similarly, Yin et al. found that under hypoxic conditions, exosomes from glioma CSCs contain miR-30b-3p, which targets RHOB, leading to decreased apoptotic ability and promoting resistance to temozolomide [112]. Yang et al. [113] reported that exosomes derived from gemcitabine-resistant pancreatic CSCs can transfer miR-210 to drug-sensitive cells, thereby inducing resistance. During this process, several drug resistance-related proteins, including MDR1, YB-1, and BCRP, are upregulated in sensitive pancreatic cancer cells, while the mTOR signaling pathway is simultaneously activated. This suggests that exosomal miR-210 from pancreatic CSCs may mediate resistance to gemcitabine in non-CSC subpopulations by increasing drug efflux. Exosomes derived from CSCs possess the ability to transfer resistance through the delivery of genetic material, enabling originally sensitive cells to acquire resistance. Lv et al. [114] found that chemotherapy-resistant breast cancer cells could transfer P-glycoprotein (P-gp) to sensitive cells via exosomes secreted by CSCs, thereby inducing chemotherapy resistance in the sensitive cells. Santos et al. [115] demonstrated experimentally that miR-155 secreted by breast CSC-derived exosomes leads to the downregulation of C/EBP-β, inhibiting the expression of TGF-β, C/EBP-β, and FOXO3a, thereby promoting EMT and chemotherapy resistance in sensitive cells.

### Exosomal ncRNAs from CSCs enhance immune suppression

The immune system effectively recognizes and eliminates foreign pathogens. Once an invader is detected, the immune system activates various biochemical processes to trigger an immune response and protect the body. However, tumor cells can evade immune surveillance and attacks by suppressing immune cell function and weakening recognition signals, thereby promoting their own growth. It has been reported that exosomes derived from CSCs can carry immune-suppressive molecules, reducing immune cell activity and facilitating immune evasion by tumor cells. For example, exosomes derived from brain CSCs contain the extracellular matrix protein tenascin-C (TNC), which, through interaction with integrin receptors α_5_β_1_ and α_v_β_6_, inhibits the mTOR signaling pathway in T cells, reducing T cell activity in co-culture and allowing tumor cells to escape immune defense [116]. Yin et al. [117] discovered that exosomes derived from CSCs are rich in the immune-suppressive protein programmed cell death ligand 1 (PD-L1). PD-L1 is highly expressed on the surface of tumor cells and binds to receptors on T cells, inhibiting T cell activation and allowing cancer cells to evade antitumor immunity. CSCs can promote the formation of immune evasion mechanisms by activating the EMT/β-catenin/STT3/PD-L1 signaling axis. In this pathway, EMT induces the transcriptional activity of β-catenin, which upregulates the expression of N-glycosyltransferase STT3. This, in turn, mediates PD-L1 N-glycosylation, stabilizing the protein structure of PD-L1 and enhancing its expression, thereby increasing the immune evasion capability of tumor cells [118]. Exosomes derived from colorectal CSCs carry miR-17-5p, which inhibits the expression of SPOP, promoting tumor cell growth and suppressing antitumor immunity in colorectal cancer by upregulating PD-L1 [119]. Additionally, CSCs can express high levels of CD47, which binds to the SIRPα receptor on macrophages, sending a “don’t eat me” signal that allows them to evade immune surveillance and clearance [120]. Furthermore, antigen-presenting cells, such as dendritic cells, process and present antigens to T cells to initiate an immune response, with antigen-processing-related transporters (TAP) playing a critical role in this process. Exosomes derived from CSCs can disrupt tumor antigen presentation, thereby reducing the immune response. Studies have shown that the expression of HLA class I or TAP molecules in CSCs is significantly lower than in non-CSCs in melanoma, glioblastoma, lung cancer, and colorectal cancer [121]. Exosomes derived from renal CSCs can induce abnormal dendritic cell differentiation, leading to an increase in immature dendritic cells and suppression of immune function [122]. Ding et al. [123] found that exosomes derived from pancreatic cancer cells, which highly express miR-212-3p, can inhibit the expression of the regulatory factor X-associated protein (RFXAP), leading to a decrease in the expression of antigen recognition molecule MHC II and inducing immune tolerance. Exosomes derived from CSCs can also promote immune evasion by tumor cells by suppressing the cytotoxic functions of immune cells. For example, NK cells typically recognize and kill tumor cells through the NKG2D receptor-ligand recognition mechanism. However, breast cancer CSCs can downregulate the expression of NKG2D ligands (MICA and MICB) via exosomes, thus weakening the recognition and killing ability of NK cells, ultimately promoting immune evasion and enhancing the metastatic potential of tumor cells [124].

Additionally, exosomes released by CSCs can mediate the exchange of information between cells and the TME, maintaining its stability, adapting to changes in the TME, and responding to these fluctuations. They can also regulate immune responses by interacting with various immune cells within the TME, weakening the immune system’s ability to recognize and clear CSCs, ultimately promoting the formation of an immune niche conducive to tumor growth and metastasis [125]. According to relevant studies, ncRNAs secreted by CSCs can act on different types of immune cells to induce and maintain an immunosuppressive microenvironment. Exosomes derived from oral squamous CSCs deliver lncRNA UCA1, which binds to miR-134 and targets LAMC2 to regulate the PI3K/AKT pathway in macrophages, a process that has been validated in both in vitro and in vivo experiments. This indicates that the exosomes promote M2 macrophage polarization, leading to an immunosuppressive microenvironment that fosters tumor malignant progression [126]. Exosomes derived from colorectal CSCs deliver miR-146a, which increases tumor-infiltrating CD66^+^ neutrophils and decreases tumor-infiltrating CD8^+^ T cells, suggesting that these exosomes may inhibit the TME [127]. Additionally, cytokines such as IL-6, VEGF, and TNF-α in the TME can directly induce tumor metastasis and tumor cell proliferation. Yang et al. found that glioma CSCs release exosomes carrying lncRNA MALAT1, which induce LPS-stimulated microglia to secrete IL-6 and TNF-α, thereby promoting tumor malignancy [128]. Exosomes released by colorectal CSCs contain a unique triphosphate RNA, which, through pattern recognition receptor interaction with the NF-κB signaling axis, induces IL-1β expression in neutrophils, extending their survival. Subsequently, neutrophils are attracted to the TME by chemokines CXCL1 and CXCL2, accelerating colorectal cancer progression [97].

### Exosomal ncRNAs from CSCs regulate tumor metabolism

Exosomal ncRNAs derived from CSCs can regulate the expression or activity of metabolic enzymes and alter the metabolic pathways of tumor cells, thereby influencing their energy supply and material metabolism, which in turn controls tumor growth and metastasis. Monocarboxylate transporters (MCT1 and MCT4) play a key role in the metabolic cooperation between tumor cells and CSCs. Curry et al. [129] discovered that in head and neck squamous cell carcinoma, tumor cells highly express MCT4, while CSCs predominantly express MCT1. This expression pattern suggests a metabolic synergy between tumor cells and CSCs. This metabolic cooperation maximizes the survival and proliferation of tumor cells. Additionally, exosomes derived from colorectal cancer can deliver specific circRNAs, such as has-circ**-**0005963 (also known as ciRS-122), to chemotherapy-sensitive cells. By acting as a “sponge” for miR-122, these exosomes upregulate the expression of pyruvate kinase M2 (PKM2), promoting glycolysis and enhancing tumor resistance to chemotherapy [130]. Exosomes secreted by breast CSCs contain circCARM1, which functions through a sponge-like mechanism to bind miR-1252-5p and regulate the expression levels of the PFKFB2 gene. This finding reveals the crucial role of circCARM1 in the glycolytic process of breast cancer cells [131]. Additionally, exosomes can regulate tumor cell metabolic pathways through other ncRNAs, such as lncRNAs. Li et al. discovered that exosomal lncRNA SNHG3 acts as a sponge for miR-330-5p, positively regulating the expression of PKM, inhibiting oxidative phosphorylation (OXPHOS), and increasing glycolysis. This metabolic reprogramming promotes the growth of breast cancer cells [132]. lncRNAs in exosomes derived from CSCs play a vital role in mediating tumor metabolic reprogramming. In exosomes secreted by lung CSCs, the highly expressed lncRNA Mir100hg targets miR-15a-5p and miR-31-5p in tumor cells, enhancing glycolytic activity and thereby increasing the tumor’s metastatic potential [133]. Similarly, in exosomes derived from melanoma CSCs, lncRNA Mir100hg enhances glycolysis and promotes tumor metastasis by regulating the expression of miR-16-5p and miR-23a-3p [134]. Zhang et al. also found that exosomal lncRNA ROLLCSC from lung cancer CSCs acts as a competitive endogenous RNA (ceRNA) for miR-5623-3p and miR-217-5p, targeting lipid metabolism pathways. This regulation enhances the plasticity of non-CSC cells and promotes tumor progression [135]. Under hypoxic conditions, exosomes from glioma CSCs highly express lncRNA H-GSC, which regulates the MZF1/c-Myc/HIF-1α axis, significantly promoting tumor cell proliferation and glycolytic metabolism in vitro [136].

## Clinical applications of exosomal ncRNAs

### Exosomal ncRNAs as potential diagnostic and prognostic biomarkers

Exosomal ncRNAs exhibit distinct expression patterns in a variety of diseases, making them potential biomarkers. In cancer, exosomal ncRNAs are considered ideal candidates for early cancer diagnosis, prognostic prediction, and disease progression monitoring due to their stability and ease of detection in bodily fluids. For example, Several articles have demonstrated that exosomes have the potential to serve as biomarkers for the early diagnosis of cancer. For instance, Marco et al. analyzed the surface proteins of serum exosomes from patients with laryngeal cancer and healthy controls, and found that exosomes can be highly specific indicators for the early diagnosis of laryngeal cancer [137]. Exosomal circSHKBP1 has been detected in the blood and is involved in the malignant progression of gastric cancer, making it a promising biomarker for diagnosing and assessing the prognosis of gastric cancer, with potential as a therapeutic target [138]. Compared to conventional gastric cancer tumor markers such as CEA, CA-199, and CA-724, exosome-derived lncRNA-GC1 offers superior diagnostic value for gastric cancer [139]. Additionally, several exosomal miRNAs (let-7A, miR-1229, miR-1246, miR-150, miR-21, miR-223, and miR-23a) have been identified as biomarkers for diagnosing colorectal cancer (CRC) [140]. Bioinformatics analysis further reveals that high expression levels of lncRNA GAS5 and miR-221 in tissues, plasma, and exosomes hold diagnostic value for colorectal cancer (CRC), impacting CRC cell proliferation, migration, and invasion, while also serving as prognostic factors for CRC [141]. Additionally, Lu et al. found that exosomal circ0048117 extracted from serum is positively correlated with the TNM staging of esophageal cancer, suggesting that it could serve as an important marker for monitoring disease progression [142]. Circulating exosomal miRNA-21 and lncRNA-ATB are negatively correlated with the overall survival of HCC patients, making them potential prognostic biomarkers for HCC [143]. Serum exosomal circ-PDE8A levels are positively correlated with lymphatic infiltration, TNM staging, and poor survival rates in pancreatic adenocarcinoma (PADC), suggesting that it could be an important prognostic biomarker for PADC [144]. Notably, studies have shown that miRNAs derived from CSC-originated exosomes in the body fluids of patients with liver cancer, lung cancer, prostate cancer, and breast cancer differ significantly from the miRNA content found in normal human fluids [145, 146]. This indicates that exosomes derived from CSCs could potentially serve as early diagnostic markers and indicators for predicting metastasis.

### Exosomal ncRNA as potential therapeutic targets in cancer

#### Targeting tumor-derived exosomal ncRNA

Exosomal-derived ncRNAs play a critical role in promoting tumor development, making them potential therapeutic targets. By inhibiting the expression or function of these ncRNAs, it is possible to effectively suppress cancer cell growth and metastasis, thereby improving treatment outcomes. AGO2, serving as a core regulatory factor in the maturation and biosynthesis of miRNAs, can, when silenced, lead to the impairment of the functional capacity of the RNA-induced silencing complex (RISC). This, in turn, disrupts the inhibitory effects of oncogenic miRNAs (such as miR-155 and miR-21) on tumor suppressor genes. Ultimately, this mechanism activates the caspase-dependent apoptotic pathway, selectively inducing apoptosis in myeloid leukemia cells [147]. Additionally, the accessory protein Vps4A plays an important role in regulating exosome release. It can inhibit the secretion of oncogenic miRNAs in hepatocellular carcinoma exosomes while promoting the secretion of tumor-suppressive miRNAs, thus significantly inhibiting tumor growth [148]. Huang et al. [149] investigated a novel anticancer peptide, PEG-SMR-Clu, which reduces the expression of ncRNAs by inhibiting exosome release. This peptide causes breast cancer cells to arrest in the G2/M phase of the cell cycle, thereby limiting breast cancer metastasis and angiogenesis to some extent. Antisense oligonucleotides (ASOs), synthetic molecules that block mRNA synthesis, can inhibit the production of pathogenic proteins or ncRNAs at the genetic level. This therapeutic approach has received approval from the FDA [150]. In a glioblastoma (GBM) xenograft model, delivering anti-miR-21 antisense oligonucleotides (antisense-ODNs) to the tumor site can block the translation of miR-21 mRNA, triggering apoptosis in tumor cells [151]. Notably, an lncRNA antisense transcript of hypoxia-inducible factor-1α (HIF-1α) is upregulated in cancer tissues. When HIF-1α is knocked out in GBM cells, exosomes regulate factors associated with angiogenesis and migration, thereby inhibiting cell growth and invasiveness in GBM [152]. Furthermore, current research has found that modifying the surface properties of exosomes or designing cell surface receptor antagonists can block the uptake pathways of ncRNAs within exosomes, thereby partially inhibiting tumor growth and progression. For example, the protein dipeptidyl peptidase 4 (DPP4), which is abundant on the surface of senescent cells, can partially prevent the uptake of exosomes carrying ncRNAs. Further experiments have shown that DPP4 antibodies or inhibitors can significantly increase the efficiency of exosome uptake, providing a new avenue for tumor therapy [153].

#### Targeting CSC-derived exosomal ncRNA

CSCs can transfer stem-like characteristics to non-CSCs via exosomes, thereby maintaining CSC homeostasis and promoting tumor progression. Therefore, targeting CSC-derived exosomal ncRNAs can disrupt signaling between CSCs and the TME or distant cells, providing an innovative therapeutic strategy to overcome treatment resistance. Current research primarily focuses on reducing the secretion of exosomes from CSCs using specific drugs or inhibitors, thereby diminishing their impact on surrounding cells and the microenvironment, which in turn reduces tumor growth and metastatic potential. Studies have shown that low-dose IFN-α treatment can reduce exosome secretion in malignant melanoma CSCs, significantly decreasing their tumorigenic potential and stemness. Sequencing analysis also revealed that the expression of certain oncogenic miRNAs in CSC-derived exosomes, such as miR-98-5p, miR-191, miR-744-3p, and let-7e-3p, was downregulated [154]. Additionally, the traditional Chinese medicine *Cinnamomum cassia* (niu zhang zhi) has gained significant attention for its multiple functions, including anti-inflammatory, antioxidant, and cancer-preventive properties. Research has shown that *Cinnamomum cassia* can significantly downregulate the expression of over 20 miRNAs, including hsa-miR-21 and hsa-miR-375, thereby inhibiting the growth and proliferation of brain CSCs and breast CSCs. As a result, this herb could serve as an effective adjunct to radiation or chemotherapy, enhancing antitumor efficacy while also mitigating chemotherapy-related side effects to some extent [155]. Chen et al. found that treatment with ovatodiolide could sensitize CSCs to cisplatin by reducing various substances, including miR-21-5p, in CSC-derived exosomes, thus inhibiting tumorigenesis in oral squamous cell carcinoma at its origin [106]. Erismodegib, a Phase III drug for treating medulloblastoma, can induce glioma stem-like cell apoptosis by inhibiting miR-21 expression and suppressing EMT by upregulating miR-128 in GSCs [156]. In addition to directly targeting exosomal ncRNAs for therapy, another approach involves mediating the signaling pathways that regulate exosome release to inhibit their secretion, thereby suppressing the CSC phenotype. Lin et al. found that inhibiting EP4 signaling in cancer xenografts reduced the number of exosomes secreted by CSCs, leading to a significant decrease in the CSC population and a marked reduction in CSC intrinsic resistance to conventional chemotherapy drugs [157]. After treatment with docetaxel, the expression of miR-9-5p, miR-195-5p, and miR-203a-3p in breast cancer exosomes increased, enhancing the CSC phenotype and cell resistance. By blocking the exosomal miRNA-ONECUT2 axis, CSC stemness and tumorigenic potential were weakened, thereby reducing chemotherapy resistance in breast cancer [158]. Additionally, aspirin has been shown to suppress exosome release under hypoxic conditions via the HIF-1α/COX-2 pathway, thereby reducing CSC numbers and inhibiting tumor cell proliferation, migration, and angiogenesis [159].

### Exosomal ncRNA as Drug Carriers For Targeted Delivery

As a natural delivery vehicle, exosomes have the ideal nanoscale size and stable membrane structure, allowing them to effectively carry and protect drugs while preventing degradation and inactivation. In recent years, exosomes have gradually been applied in the field of drug delivery due to their relatively safe and easily modifiable physicochemical properties, scalability, low cost, and strong loading capacity [160]. Non-coding RNAs, as gene therapy carriers for various diseases (including cancer), have gained significant attention for their potential. Through engineering techniques, exogenous tumor-suppressive ncRNAs can be loaded into exosomes and delivered to tumor sites, effectively inhibiting tumor progression to some extent [161]. CSCs exhibit significant resistance to conventional chemotherapy and radiation due to their unique dormant characteristics, efficient DNA repair mechanisms, and high expression of drug efflux transporters, leading to tumor recurrence and distant metastasis. However, exosomes, with their good biocompatibility and low immunogenicity, can deliver anticancer drugs to CSCs under specific targeting molecular interactions, directly intervening in the root cause of treatment resistance [8].

#### Exosomal targeted delivery to tumor cells

Using exosomes as carriers to precisely deliver therapeutic ncRNAs to tumor cells can effectively enhance cancer treatment efficacy while reducing treatment-related side effects, offering significant clinical application potential and prospects. Thomas et al. found that loading miR-186 into exosomes and delivering them to a neuroblastoma orthotopic mouse model resulted in reduced tumor burden and increased survival rates, demonstrating the therapeutic potential of exosomal delivery of miR-186 in neuroblastoma [162]. Ding et al. [163] transfected miR-145-5p into exosomes and targeted their delivery to pancreatic cancer cells. They found that this approach significantly increased tumor cell apoptosis and notably inhibited tumor growth. Jing et al. [164] used electroporation to load miR-499 into exosomes derived from BM-MSCs. After delivering the exosomes to the tumor site, they observed a significant inhibition of endometrial cancer cell proliferation, EC tube formation, and suppression of tumor growth and angiogenesis. lncRNAs are also commonly used as carriers for exosomal delivery. For example, lncRNA MEG3 has shown significant inhibitory effects on osteosarcoma. Researchers engineered exosomes by modifying them with c(RGDyK) and loading them with MEG3, achieving effective delivery to osteosarcoma cells both in vitro and in vivo [165].

Additionally, using exosomes as carriers to deliver agents that suppress the expression of oncogenic ncRNAs is another effective strategy for cancer treatment. A commonly used approach is gene editing technologies, such as the CRISPR-Cas9 system or RNA interference (RNAi), which can directly target and knock out or inhibit the expression of specific ncRNAs to achieve gene silencing [166]. CRISPR/Cas9 is highly efficient in locating and editing target genes, making it a powerful therapeutic tool. However, achieving specific and safe delivery in vivo remains a significant challenge. When CRISPR/Cas9 is loaded into exosomes and targeted to tumor sites, it can notably overcome these limitations and enhance tumor apoptosis to some extent [167]. LncRNA H91 has been shown to promote colorectal cancer (CRC) progression by regulating the expression of HNRNPK. Gao et al. packaged small interfering RNA (siRNA) targeting H91 into exosomes and delivered them to colorectal cancer cells, resulting in a significant inhibition of tumor growth, validating the effectiveness of using exosomes for targeted siRNA delivery to suppress ncRNA expression [168]. Another study found that the lncRNA HISLA enhances aerobic glycolysis and resistance to apoptosis in breast cancer cells by inhibiting the hydroxylation and degradation of HIF-1α. By using aptamers to load siRNA conjugates into exosomes, HISLA expression was specifically silenced, stabilizing HIF-1α expression. This could represent a viable method to inhibit tumor metabolic reprogramming [169]. Additionally, inhibitors or antagonists targeting specific ncRNAs can also suppress their expression by affecting their transcription, processing, or stability. Wang et al. loaded miR-21 inhibitors into engineered exosomes and delivered them to gastric cancer (GC) cells. Compared to traditional transfection methods, this approach showed a more significant inhibitory effect with lower cytotoxicity [170]. Rajendran et al. [171] loaded anti-miRNA-21 and anti-miRNA-10b into urokinase-type plasminogen activator-engineered exosomes (uPA-eEVs), which significantly enhanced tumor-targeting affinity. This treatment, in combination with low-dose doxorubicin, exhibited a synergistic antitumor effect, effectively inhibiting breast cancer growth and reducing lung metastasis. Moreover, transfecting miRNA-BART1-5p antagonists into exosomes for targeted therapy significantly inhibited angiogenesis in tumor tissues and induced cell apoptosis [172]. Overall, these studies not only confirm the immense potential of exosomes in ncRNA-targeted therapy but also open new directions and approaches in the field of cancer treatment.

#### Exosomal targeted delivery to CSCs

In recent years, CSC-targeted therapies have gained significant attention, with various new strategies such as CSC biomarker-mediated targeting, mitochondrial targeting, and pathway targeting being developed and yielding promising results. However, due to the complex microenvironment, the unique biological characteristics of CSCs, and challenges in drug delivery, therapeutic outcomes remain suboptimal. By utilizing exosome engineering techniques to target unique CSC markers, more precise targeted therapies can be achieved. EpCAM is a key marker of CSCs. Studies have shown that by engineering exosomes to specifically recognize and target liver CSCs expressing EpCAM and loading them with β-catenin-specific siRNA, it is possible to effectively inhibit the Wnt/β-catenin signaling pathway, significantly suppressing CSC proliferation. This approach offers a novel therapeutic strategy for liver cancer treatment [173]. In addition, Naseri et al. [174] used electroporation to load anti-miR-142-3p genes into exosomes and deliver them to tumor sites. By inhibiting the expression or function of endogenous miR-142-3p, they interfered with the signaling pathways associated with the tumorigenicity of breast CSCs, thereby reducing the tumorigenic potential of breast cancer CSCs in vivo and suppressing malignant tumor progression. Alessia et al. [175] found that exosomes derived from human liver stem cells (HLSC) could deliver miR-145 and miR-200 to induce apoptosis in renal CSCs, reducing CSC proliferation, spheroid formation, and invasion. Taking advantage of exosomes as drug delivery carriers, Yong et al. developed engineered exosome-like porous silicon nanoparticles (PSiNPs) as drug carriers for targeted cancer therapy. These nanoparticles exhibit high biocompatibility, reduce immune rejection, and their porous structure allows for slow drug release, enhancing therapeutic efficacy. Further experiments showed that PSiNPs demonstrated significant cellular uptake and cytotoxicity in a wide range of cancer cells and CSCs [176]. In conclusion, exosome-based targeted strategies for CSCs hold great promise for improving issues related to tumor recurrence, drug resistance, and metastasis, offering the potential for personalized treatment.

**Fig. 3:**
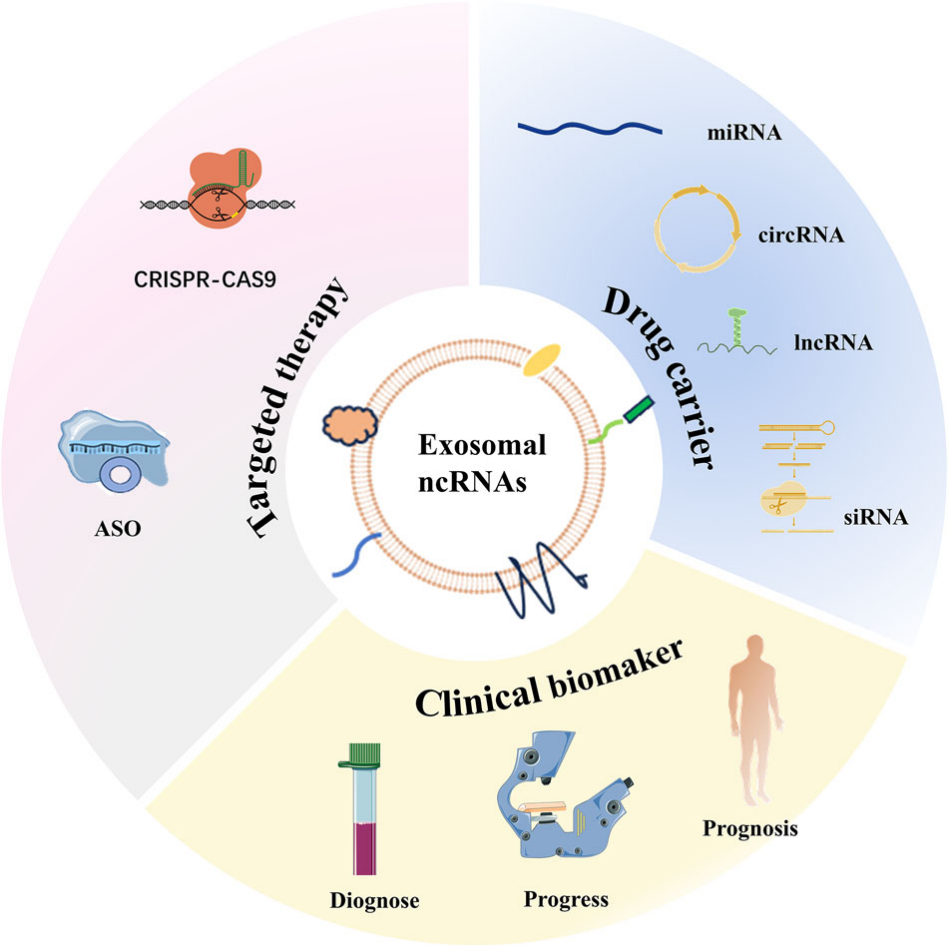
Potential clinical applications of exosomal ncRNAs. They can serve as potential biomarkers; certain oncogenic exosomal ncRNAs may also be targeted for therapeutic purposes, and exosomes can be used as delivery vehicles for ncRNA-based therapies.

As previously mentioned, the value of exosomal ncRNAs in clinical therapy primarily manifests in three aspects: (1) serving as non-invasive biomarkers for early diagnosis, prognostic evaluation, and monitoring of treatment response; (2) acting as targets for therapeutic intervention by silencing or inhibiting oncogenic exosomal ncRNAs; (3) functioning as delivery vehicles for therapeutic ncRNAs, leveraging their advantages in biocompatibility and targeting. Figure 3 visually summarizes these three crucial directions of clinical applications for exosomal ncRNAs.

## Summary and outlook

The state-of-the-art studies have demonstrated that ncRNAs play a pivotal role in mediating bidirectional communication between cancer cells and CSCs. Tumor cells and other cellular components within the TME deliver specific exosomal ncRNAs into CSCs, reinforcing their stemness and maintaining their homeostasis. On the contrary, CSC-derived exosomal ncRNAs reciprocally drive metastatic progression, angiogenesis, and chemoresistance of the tumor tissue. In this work, it is unequivocally delineated the central regulatory role of exosomal ncRNAs in the tumor progression process and specifically, it is deciphered the mechanistic contributions of the ncRNAs to CSC-driven tumor aggressiveness--a conceptual advance bridging the knowledge gap in malignancy evolution. These findings position ncRNAs as promising therapeutic targets with dual diagnostic-prognostic utility, offering novel strategies for chemoresistance reversal and precision oncology. Furthermore, the discovery that CSCs make use of exosomal ncRNA-mediated pathways to evade immune surveillance and clearance provides a mechanistic foundation for developing CSC-targeted immunotherapies, highlighting previously underappreciated vulnerabilities in tumor immunoediting.

While targeting CSCs and their exosomal ncRNAs holds transformative potential in oncology, five interconnected challenges impede clinical translation: (1) The inherent complexity of multifactorial carcinogenesis limits therapeutic efficacy depending on exosomal ncRNA alone, which may be exacerbated by stage-specific exosomal ncRNA expression dynamics that compromise treatment adaptability, Hence, it is essential to construct a dynamic, multi-omics-based model that integrates data from multiple levels to develop personalized, targeted treatment plans for patients (taking into account factors such as tumor type, stage, genetic mutations, etc.); (2) Despite exosomal functions being source cell- and cargo-dependent, critical gaps persist in understanding ncRNA sorting mechanisms, exosome biogenesis/release kinetics, and recipient cell uptake specificity, compounded by variable in vivo biodistribution patterns requiring systematic mapping of exosome trafficking-behavior relationships; (3) Technical barriers including non-standardized isolation protocols, production scalability issues, and functional heterogeneity undermining liquid biopsy reliability demand standardized purification pipelines integrated with microfluidic/affinity chromatography platforms and nanoflow cytometry for subpopulation resolution; (4) Suboptimal delivery efficiency and CSC specificity, despite genetic engineering technology advances, remain constrained by shared stem cell signaling pathways, warranting organoid/PDTX model-optimized targeting strategies; (5) Insufficient long-term safety validation of engineered exosomes, despite preclinical promise, underscores the imperative for rigorous clinical trials assessing stability, immunogenicity, and context-dependent risks. Currently, the advancements achieved in engineered exosome technology, as well as combination therapy strategies targeting CSCs, are gradually approaching the critical threshold for clinical testing. By delving deeply into the elucidation of underlying mechanisms and consistently promoting technological innovation, these research endeavors hold significant promise for driving breakthrough developments in the field of precision oncology.
